# Regional Therapy Approaches for Gastric Cancer with Limited Peritoneal Disease

**DOI:** 10.1007/s12029-023-00994-5

**Published:** 2024-01-26

**Authors:** Amy Y. Li, Shaina Sedighim, Fatemeh Tajik, Aaqil M. Khan, Vinodh K. Radhakrishnan, Farshid Dayyani, Maheswari Senthil

**Affiliations:** 1 Department of Surgery, University of California, Irvine, 3800 Chapman Ave, Ste 7400, 92868 Orange, CA USA; 2 Department of Medicine, University of California, Irvine, Orange, USA

**Keywords:** Gastric adenocarcinoma, Peritoneal carcinomatosis, Intraperitoneal chemotherapy, Heated intraperitoneal chemotherapy, Normothermic intraperitoneal chemotherapy, Pressurized intraperitoneal aerosolized chemotherapy

## Abstract

**Purpose:**

Despite advances in systemic therapy, outcomes of patients with gastric cancer (GC) peritoneal carcinomatosis (PC) remain poor, in part because of poor penetrance of systemic therapy into peritoneal metastasis due to the plasma-peritoneal barrier and anarchic intra-tumoral circulation. Hence, regional treatment approach with administration of chemotherapy directly into the peritoneal cavity (intraperitoneal, IP) under various conditions, combined with or without cytoreductive surgery (CRS) has remained an area of significant research interest. The purpose of this review is to provide high-level evidence for regional treatment approaches in the management of GCPC with limited peritoneal disease.

**Methods:**

A review of the current literature and ongoing clinical trials for regional IP therapies for GCPC was performed. Studies included in this review comprise of phase III randomized controlled trials, non-randomized phase II studies, high-impact retrospective studies, and active ongoing clinical trials for each available IP modality.

**Results:**

The three common IP approaches are heated intraperitoneal chemotherapy (HIPEC), normothermic intraperitoneal chemotherapy (NIPEC) and more recently introduced, pressurized intraperitoneal aerosolized chemotherapy (PIPAC). These IP approaches have been combined with systemic therapy and/or CRS with varying degrees of promising results, demonstrating evidence of improvements in survival rates and peritoneal disease control. Patient selection, optimization of systemic therapy, and completeness of cytoreduction have emerged as major factors influencing the design of contemporary and ongoing trials.

**Conclusion:**

IP chemotherapy has a clear role in the management of patients with GCPC, and when combined with CRS in appropriately selected patients has the potential to significantly improve survival. Ongoing and upcoming IP therapy clinical trials hold great promise to shape the treatment paradigm for GCPC.

## Introduction and Background

Gastric cancer (GC) is the fifth most common cancer and fourth leading cause of cancer deaths worldwide in 2020 [1]. In the USA, an estimated 26,500 new gastric cancer cases will be diagnosed and 11,130 people will die due to this cancer in 2023 [2]. The observed high mortality is due to the high incidence of metastasis, particularly peritoneal carcinomatosis (PC). Incidence of PC in gastric cancer is about 30% at the time of initial presentation [3–5] and 15–52% at the time of recurrence [3, 6–9]. In many patients, the peritoneum is the only site of metastatic disease, and has shown association with diffuse type, signet ring histology, and poorly differentiated GC [3, 10]. The survival for patients with PC is dismal, with median overall survival (OS) of 4–10 months [3, 6, 11, 12]. Recent randomized phase III trials in metastatic gastric cancer have shown that combining targeted therapies or immune checkpoint inhibitors with systemic chemotherapy is associated with improved survival compared to systemic chemotherapy alone. However, these benefits are not uniform in all patients, and at least in part dependent on the sites of metastatic disease [3].

In the ATTRACTION-4 study, a Japanese study that tested the combination of capecitabine or S-1 + oxaliplatin with or without nivolumab, the subgroup analysis of patients with PC showed no significant benefit in progression-free survival (PFS) (7.46 vs. 8.57 months, hazard ratio (HR) 1.04), or OS (13.67 vs.15.77, HR 1.20) [13]. Since PC is often associated with signet ring and diffuse gastric cancer, survival outcomes of these specific subgroups in the more recent targeted therapy clinical trials are instructive with the caveat that these studies are not powered to detect differences in the subgroups. In CHECKMATE 649, a large, randomized study that tested the benefits of adding Nivolumab to mFOLFOX6, the OS for patients with signet ring cancers even in the PD-L1 combined positive score (CPS) ≥ 5 group was 12.1 months for the experimental group and 9.0 months for the systemic chemotherapy alone group (HR 0.71 (0.49–1.03)) [14]. This study highlights the poor survival of merely 12 months even with addition of checkpoint inhibitor to systemic chemotherapy. Similarly, in both SPOLTLIGHT and GLOW (Zolbetuximab + mFOLFOX6 or CAPEOX vs chemotherapy alone), the OS for patients with diffuse gastric cancer was not significantly different between the two arms in prespecified subgroup analysis (SPOTLIGHT HR 0.76 (0.51–1.13) and GLOW HR 0.73 (0.49–1.07)) [15, 16]. Although the FLOT regimen is currently not used as often in metastatic gastric cancer, it is important to note that in the FLOT3 study, the median OS of the group that comprised majority of patients with PC was 10 months [12]. These contemporary studies highlight the poorer outcomes for metastatic GC, in particular, signet ring/diffuse subtypes. They emphasize the need for better treatment strategies in addition to the incremental benefits seen with targeted therapies [17].

All of the aforementioned data suggests that systemic chemotherapy alone may not be sufficient to treat peritoneal metastasis. This is supported by evidence that the plasma-peritoneal barrier and anarchic intra-tumoral circulation reduce the penetrance and effectiveness of systemic therapy in PC [17, 18]. Hence, there is an underlying pathophysiologic rationale for the need to incorporate treatment strategies that combine both systemic and regional therapies which include intraperitoneal (IP) chemotherapy and in selected patients, cytoreductive surgery (CRS). The currently available IP approaches are heated intraperitoneal chemotherapy (HIPEC), normothermic intraperitoneal chemotherapy (NIPEC), and pressurized intraperitoneal aerosolized chemotherapy (PIPAC). The main advantages of IP therapy are the direct exposure of peritoneal metastases to high concentrations of chemotherapy along with limited systemic toxicity. CRS, or complete removal of all viable, gross disease, aids in obtaining macroscopic control of the disease and can be performed in conjunction with systemic and regional IP therapies in selected patients. As such, patient selection based on burden of disease, response to systemic and IP therapies, and feasibility of complete cytoreduction is crucial to achieve improvement of survival beyond what is currently possible with systemic and targeted therapies.

This review summarizes the best available evidence for the various IP therapies, the ongoing clinical trials, the importance of patient selection, and the application of the evidence in the management of patients with limited GC peritoneal metastasis.

## Heated Intraperitoneal Chemotherapy (HIPEC)

The rationale for HIPEC is based on the evidence from pre-clinical and clinical studies that showed heat-induced augmentation of cytotoxic effects of specific chemotherapeutic agents as well as increased depth of tissue penetration [19]. HIPEC is performed under general anesthesia in the operating room either by itself or as an adjunct to CRS. The most common agents used for HIPEC in GCPC are cisplatin and mitomycin C (MMC). The duration of HIPEC varies between 60 and 90 min. The treatment costs, need for surgical procedure, OR equipment, logistics, and training required for HIPEC are important factors to consider when comparing various IP approaches.

### CRS vs CRS/HIPEC Randomized Clinical Trials

There have been only two phase III HIPEC clinical trials in GCPC, both testing the benefit of adding HIPEC to CRS compared to CRS alone (Table 1).

**Table 1:** Completed randomized and non-randomized clinical trials of hyperthermic intraperitoneal chemotherapy (HIPEC) and cytoreductive surgery (CRS) in gastric cancer peritoneal carcinomatosis

Clinical Trial	Country	Study Design (size)	Study arms	Outcomes/Endpoints
Completed Randomized Clinical Trials for CRS/HIPEC				
Yang et al. [20] (2011)	China, single center	Phase III (n = 68)	Control: CRS alone (n = 34) Study: CRS/HIPEC with IP cisplatin/MMC (n = 34)	Median OS: CRS/HIPEC vs CRS alone–11.0 months vs 6.5 months; p = 0.046 Median OS for synchronous PC: CRS/HIPEC vs CRS alone–12.0 months vs 6.5 months (p = 0.029) No difference in OS for low PCI < 20 vs high PCI ≥ 20, p = 0.464
GASTRIPEC-I [21] Rau et al. (2023)	Germany, multicenter	Phase III (n = 50)	Control: CRS alone (n = 22) Study: CRS/HIPEC with IP cisplatin/MMC (n = 28)	Median OS: 14.9 months in both groups (p = 0.165) PFS: CRS/HIPEC vs CRS alone–7.1 months vs 3.5 months; p = 0.047 Trial closed early due to poor accrual. 55 patients progressed or died receiving NAC
Completed Nonrandomized Clinical Trials in CRS/HIPEC				
PERISCOPE I [22] van der Kaaij et al. (2020)	Netherlands, multicenter	Phase I-II (n = 25)	CRS + HIPEC (IP oxaliplatin) + NIPEC (IP docetaxel)	Safety and feasibility study Serious adverse events: 68% Reoperation: 16% Mortality: 8%
Badgwell et al. [24] (2017)	United States, single center	Phase II (n = 19)	Laparoscopic iterative HIPEC (IP cisplatin/MMC) At restaging, if converted to negative peritoneal cytology, then proceed with gastrectomy	Median OS: 30.2 months from dx, 20.3 months from first laparoscopy Median OS after gastrectomy: 29 months Complete histologic response: 36.8% (n = 7), with 26.3% (n = 5) proceeding to definitive resection
Badgwell et al. [25] (2021)	United States, single center	Phase II (n = 20)	CRS/HIPEC (IP cisplatin/MMC) after neoadjuvant laparoscopic HIPEC	Median OS: 22.1 months from dx, 16.1 months from CRS/HIPEC Severe morbidity: 25% No mortalities
Ongoing Clinical Trials for CRS/HIPEC				
GASTRICHIP [28, 29] Glehen et al. NCT01882933	France, multicenter (2013–2025)	Phase III 1:1 randomization intraoperatively after CRS (n = 367)	Control: CRS (gastrectomy, D1-D2 lymphadenectomy) Study arm: CRS (gastrectomy, D1-D2 lymphadenectomy) + HIPEC (IP oxaliplatin)	Primary: 5-yr OS Secondary: 3- and 5-yr RFS, site of recurrence, morbidity, QoL Ancillary: incidence of positive peritoneal cytology pre- and post-gastrectomy
PERISCOPE II [23, 31] van Sandick et al. NCT03348150	Netherlands, multicenter (2017–2029)	Phase III 1:1 randomization (n = 182)	Control: Palliative SC only Study: CRS (gastrectomy, D2 lymphadenectomy) + HIPEC (IP oxaliplatin) + NIPEC (docetaxel)	Primary: 5-yr OS Secondary: 5-yr RFS, treatment toxicity, cost-effectiveness
ROBO-CHIP [73] Grotz et al. NCT05753306	United States, single center (2023–2026)	Phase II, nonrandomized (n = 20)	Single arm: Robot-assisted gastrectomy + HIPEC (IP cisplatin/docetaxel)	Primary: hospital LOS, 30-day readmission rate, 30-day morbidity Secondary: 5-yr DFS, 5-year IP RFS, 5-year OS, open conversion rate, opioid use, pain scores, EBL, operative time
Davis et al. [32, 33] NCT03092518	United States, single center (2017–2027) Active, not recruiting	Phase II, nonrandomized (n = 27)	Single arm: CRS (gastrectomy, D2 lymphadenectomy) + HIPEC (IP cisplatin/MMC)	Primary: Median OS Secondary: IP PFS, distant (extraperitoneal) DFS, treatment morbidity

*CRS* cytoreductive surgery, *HIPEC* heated intraperitoneal chemotherapy, *IP* intraperitoneal, *MMC* mitomycin C, *OS* overall survival, *PC* peritoneal carcinomatosis, *PCI* peritoneal cancer index, *vs* versus, *PFS* progression-free survival, *NAC* neoadjuvant chemotherapy, *NIPEC* normothermic intraperitoneal chemotherapy, *dx* diagnosis, *yr* year, *RFS* recurrence-free survival, *QoL* quality-of-life, *LOS* length of stay, *DFS* disease-free survival, *EBL* estimated blood loss

In 2011, Yang et al. conducted one of the first randomized phase III clinical trials that evaluated the survival benefit of adding HIPEC to CRS in GCPC. In this study, patients with GCPC (*n* = 68) were randomized 1:1 to either CRS/HIPEC (CRS-H) with IP cisplatin (120 mg) and MMC (30 mg) vs CRS alone (CRS-A) [20]. This was a single institution study, and the principal investigator was the main surgeon for all CRS procedures. Median peritoneal cancer index (PCI) in both groups was 15 (range 2–36 in CRS-H vs. 3–23 in CRS-A). PCI of 20 was used as a cutoff for low vs. high burden disease. At a median follow up of 32 months, 97.1% of patients in the CRS-A group and 85.3% of patients in the CRS/HIPEC had died due to disease, and the majority of the patients died due to abdominal recurrence. Although the authors reported an improved median OS from 6.5 months for CRS-A to 11.0 months with the addition of HIPEC (*p* = 0.046), the survival for both groups were extremely poor. Additionally, there was no difference in OS for patients with low PCI < 20 (10.2 vs. 10.5 months, *p* = 0.464). There are several reasons that could have led to this poor survival. First, only 59% of patients in both arms had complete cytoreduction. This observation is particularly relevant as completeness of cytoreduction (CC) 0–1 was independently associated with improved survival on multivariable analysis (HR = 2.8, 95% confidence interval (CI) 1.405–5.556, *p* = 0.003). Second, administration of systemic chemotherapy was not standardized and there was no clear minimum required duration of perioperative systemic therapy, resulting in significant variability among patients. While the study was underpowered and survival was quite low in this study, this is the first randomized controlled trial (RCT) to demonstrate any survival benefit with CRS/HIPEC for GCPC. The results should be interpreted with caution given the wide range of PCI, low percentage of complete cytoreduction, and lack of standardization of systemic chemotherapy likely affecting the survival analysis.

Now, 12 years from the publication of the first RCT of CRS/HIPEC in GCPC, the results of the German RCT, GASTRIPEC-I, were recently published. GASTRIPEC-I is a multicenter phase III clinical trial that evaluated CRS/HIPEC (CRS-H) compared to CRS alone (CRS-A) in GCPC [21]. The HIPEC regimen consisted of cisplatin (75 mg/m^2^) and MMC (15 mg/m^2^) for 60 min. Primary endpoint was OS, and secondary endpoints were peritoneal PFS, other distant metastasis–free survival (MFS), and safety. The trial closed early due to poor accrual and the results of the 105 patients accrued between March 2014 and June 2018 were recently reported. Fifty-three patients were randomized to CRS-A and 52 patients to CRS-H. Unfortunately, 55 patients progressed or died while receiving neoadjuvant chemotherapy. As such, only 22 (41%) in the CRS-A arm and 28 (54%) in the CRS-H arm underwent surgery. The median PCI in both groups was 5 (range CRS-H 2–11; CRS-A 3–8). There was no difference in OS between the two groups (14.9 vs. 14.9 months, *p* = 0.165). The PFS (peritoneal) was 7.1 months (95% CI 3.7 to 10.5) in the CRS-H group compared to 3.5 months (95% CI 3.0–7.0; *p* = 0.047) in the CRS-A group. Though the authors had defined disease burden based on PCI (low (≤ 6), moderate (7–13), or high (> 13)), subgroup analysis based on PCI could not be performed due to low statistical power.

Although the GASTRIPEC study showed improved peritoneal disease control with the addition of HIPEC, it also has brought to light several important points to consider in the design of GCPC trials. Nearly half the patients enrolled in the study had disease progression or death during the preoperative systemic chemotherapy, underscoring the need for selection after a certain duration of systemic therapy to avoid loss of significant number of enrolled patients prior to initiating the study treatment. In the patients who underwent surgery, complete cytoreduction was achieved in only 47.4% of patients, and 31.6% of patients had unresectable disease [21]. These results emphasize the importance of patient selection for CRS as complete cytoreduction is a crucial factor for improved survival in GCPC and the need to optimize the most effective upfront systemic regimen. Finally, the OS for both groups was 14.9 months and has to be taken in the context of the OS reported in the recent first-line systemic therapy trials in metastatic GC (Table 2).

**Table 2:** Comparison of survival of GASTRIPEC to first-line systemic therapy trials in metastatic gastric cancer

Trial	Target	Experimental	Standard	PFS (months)	OS (months)
GASTRIPEC [21]		EOX or FLOT + CRS + HIPEC	EOX or FLOT + CRS	7.1 vs. 3.7	14.9 vs. 14.9
Checkmate 649 [71]	PD-L1 CPS score > 1	FOLFOX + Nivo	FOLFOX	7.5 vs.6.9	14 vs.11.3
SPOTLIGHT [15]	Claudin18.2	FOLFOX + Zolbetuximab	FOLFOX	10.6 vs.8.7	18.2 vs.15.5
GLOW [16]	Claudin18.2	CAPOX + Zolbetuximab	CAPOX	8.2 vs.6.8	14.3 vs.12.1
ToGA [72]	HER2 +	5-FU/capecitabine + Cisplatin + Trastuzumab	5-FU/capecitabine + Cisplatin	6.7 vs.5.5	13.8 vs.11.1

*PFS* progression-free survival, *OS* overall survival, *EOX* epirubicin/oxaliplatin/xeloda, *FLOT*, 5-fluorouracil/leucovorin/oxaliplatin/docetaxel, *CRS* cytoreductive surgery, *HIPEC* heated intraperitoneal chemotherapy, *FOLFOX* leucovorin/5-fluorouracil/oxaliplatin, *Nivo* nivolumab, *PD-L1* programmed death-ligand 1, *CPS* combined positive score, *CAPOX* capecitabine/oxaliplatin, *5-FU* 5-fluorouracil, *HER2* human epidermal growth factor receptor 2

### CRS/HIPEC Non-randomized Clinical Trials

There have been some important single arm phase II clinical trials that have been conducted to evaluate the safety, feasibility, and efficacy of CRS/HIPEC, particularly in patients with limited peritoneal disease (Table 3).

**Table 3:** Completed and ongoing randomized and non-randomized clinical trials of normothermic intraperitoneal chemotherapy (NIPEC) in gastric cancer peritoneal carcinomatosis

Clinical Trial	Country	Study Design (n)	Study arms	Outcomes
Completed Randomized Clinical Trials for NIPEC				
PHOENIX-GC [43] Ishigami et al. (2018)	Japan, multicenter	Phase III (n = 164)	Control: SC alone (S-1 + IV PTX) (n = 50) Study: SC + NIPEC (S-1 + IV/IP PTX) (n = 114)	Median OS: SC + NIPEC vs SC alone–17.7 months vs 15.2; p = 0.080 3-year OS: SC + NIPEC vs SC alone–21.9% vs 6.0%; p < 0.05
INPACT [47] Takahashi et al. (2018)	Japan, multicenter	Phase II (n = 83)	Control: Postoperative IV PTX weekly × 7, followed by SC (n = 44) Study arm: Postoperative IP PTX weekly × 7, followed by SC (n = 39)	Median OS: IP PTX vs IV PTX–42.3 vs 37.7 months; p = 0.63 Media PFS: IP PTX vs IV PTX–20.1 vs 22.8 months; p = 0.62 No survival difference between IV vs IP PTX
Completed Nonrandomized Clinical Trials in NIPEC				
Chia et al. [48] (2022)	Singapore, single center	Phase II, case control matched with retrospective cohort (n = 44)	SC (CAPOX) + NIPEC (IP paclitaxel)	Median OS: SC + NIPEC vs matched SC pts–14.6 months vs 10.6 months; p = 0.002 PFS: SC + NIPEC vs matched SC pts–9.5 months vs 4.4 months; p < 0.001 IP conversion to surgery: 36.1% (n = 13/36); median OS: 24.2 months; 1-year OS: 84.6%
Yamaguchi et al. [45] (2013)	Japan, single center	Phase II (n = 35)	S-1 + IV/IP paclitaxel At restaging, CRS with gastrectomy if cytology negative	Median OS: 17.6 months 1-year OS: 77.1% (p < 0.05); 2-year OS: 44.8% (p < 0.05) PCI ≥ 20 trended toward lower survival (13.4 months, p = NS) Objective response rate: 71% (n = 5/7) Peritoneal cytology conversion to negative: 97% (n = 28/29), surgery performed in 21 patients
Shinkai et al. [50] (2018)	Japan, single center	Phase II (n = 17)	SC (S-1 + IV paclitaxel/cisplatin) + IP paclitaxel)	Median OS: 23.9 months 1-year OS: 82.4%; 2-year OS: 47.1%; 5-year OS: 23.5% (p < 0.05) RECIST tumor response: 70.5% (n = 12/17) Complete PC response: 64.7% (n = 11/17), underwent surgery; median OS: 32.8 months vs 12.9 months (PC still present after treatment)
Kobayashi et al. [49] (2023)	Japan, multicenter	Phase II (n = 53)	S-1 + IV/IP paclitaxel	Median OS: 19.4 months 1-year PFS: 57% Histologic response rate: 64% (n = 23/36), with 14 patients (26%) undergoing gastrectomy
Ongoing Clinical Trials for NIPEC				
STOPGAP [51, 52] Senthil et al. NCT04762953	United States, single center (2021–2025)	Phase II, nonrandomized (n = 35)	Single arm: IV paclitaxel, 5-FU and leucovorin + IP paclitaxel At restaging, if PCI ≤ 10, CRS + HIPEC (IP cisplatin/MMC)	Primary: 1-yr PFS Secondary: OS, QoL Ancillary: plasma exosomal gene signature associated with response
Dias et al. [53, 54] NCT05541146	Brazil, single center (2022–2025)	Phase II, nonrandomized (n = 30)	Single arm: SC + IP paclitaxel × 4 cycles After 4 cycles, at restaging, if peritoneal response, then undergo gastrectomy	Primary: Complete peritoneal response Secondary: PFS, OS, safety and tolerability, treatment morbidity, diagnostic accuracy of peritoneal lavage evaluation methods
IPLUS [55, 56] Kim et al. NCT03618758	South Korea, single center (2018–2023)	Phase I-II, nonrandomized (n = 43)	Single arm: SC (mFOLFOX) + IP paclitaxel × 3 cycles At restaging, if peritoneal response, then undergo conversion surgery	Primary: 1-yr OS Secondary: 1-yr PFS, toxicity, tumor response, conversion surgery rate
David et al. [74, 75] NCT04034251	United States, single center (2020–2027) Active, not recruiting	Phase II, nonrandomized (n = 12)	Single arm: IV/IP paclitaxel + oral capecitabine	Primary: PFS Secondary: IP PFS, distant (extraperitoneal) DFS, histopathologic response, treatment morbidity, OS Ancillary: correlate response with proteogenomic subtyping

*NIPEC* normothermic intraperitoneal chemotherapy, *SC* systemic chemotherapy, *IP* intraperitoneal, *IV* intravenous, *PTX* paclitaxel, *OS* overall survival, *vs* versus, *PFS* progression-free survival, *pts* patients, *CAPOX* capecitabine/oxaliplatin, *PCI* peritoneal cancer index, *CRS* cytoreductive surgery, *RECIST* Response Evaluation Criteria in Solid Tumors, *PC* peritoneal carcinomatosis, *5-FU* 5-fluorouracil, *yr* year, *QoL* quality-of-life, *mFOLFOX* leucovorin/5-fluorouracil/oxaliplatin, *DFS* disease-free survival

The PERISCOPE I study, conducted in the Netherlands, was a multicenter phase I-II trial designed to assess the safety of fixed-dose oxaliplatin for HIPEC (460 mg/m^2^) for 30 min combined with CRS after systemic therapy in patients with limited GCPC [22]. The study also explored the maximum tolerated dose of normothermic docetaxel combined with HIPEC utilizing escalating doses of docetaxel (0 mg/m^2^, 50 mg/m^2^, and 75 mg/m^2^).

In this study, patients with locally advanced (cT3–T4a) GC with either positive peritoneal cytology (cyt +) or limited PC, defined as peritoneal lesions limited to the upper abdominal cavity with no more than one location in the lower abdomen, were treated with three to four cycles of systemic chemotherapy followed by gastrectomy/CRS and oxaliplatin HIPEC for 30 min, followed by normothermic docetaxel for 90 min [22]. In total, between 2014 and 2017, 37 patients were enrolled and 25 completed the full study protocol. The median PCI was 2 (range 0–6). Serious adverse events were reported in 68% of patients, reoperation was required in 16% of patients, and treatment-related mortality occurred in two patients (8%) in dose level 3. The study determined the fixed-dose oxaliplatin is safe to be combined with CRS, and 50 mg/m^2^ of docetaxel is the acceptable dose to be combined with HIPEC. The ongoing PERISCOPE II, a continuation phase III trial, is based on the results of PERISCOPE I [23] and is discussed later in this paper.

### Iterative HIPEC — Phase II Studies

At the MD Anderson Cancer Center in the USA, Badgwell et al. recently conducted two consecutive phase II studies. The first is a single-center study that evaluated the safety, feasibility, and efficacy of iterative laparoscopic HIPEC after systemic chemotherapy in GC patients with either cyt + disease or limited PC [24]. Laparoscopic HIPEC was performed with cisplatin 200 mg and MMC 30 mg for 60 min and could be repeated every 3 weeks for up to five times. Gastrectomy was offered to patients who had complete resolution of peritoneal disease as determined by conversion to negative cytology, no laparoscopic evidence of carcinomatosis, and no imaging evidence of solid organ metastases. Between 2014 and 2016, 19 patients were enrolled in the study, of which six patients had cyt + disease and 13 had image-occult PC. A total of 38 laparoscopic HIPEC procedures were performed in 19 patients, and 53% of patients received a single HIPEC treatment. The overall complication rate for HIPEC was 11%, with no 30-day mortalities. The median hospital stay was 3 days (range 2–6 days). Resolution of peritoneal disease was seen in 7/19 patients (36.8%) of which five patients elected to undergo surgery. Four of the five patient had only cyt + disease. The median OS for patients from the time of the first laparoscopic HIPEC was 20.3 months and for the five patients who underwent gastrectomy was 29 months.

In a follow-up phase II study, Badgwell et al. evaluated the survival of patients who underwent CRS/HIPEC after preoperative systemic chemotherapy and at least one laparoscopic HIPEC [25]. Of the 20 patients included in the study between 2016 and 2019, 14 patients had gross carcinomatosis and 6 had cyt + disease. The median PCI at the time of CRS/HIPEC was 2 (range 0–13). While the duration and number of iterative laparoscopic HIPEC procedures was at the discretion of the surgeon, 75% of patients underwent one HIPEC, while 25% underwent two HIPEC procedures. There were no perioperative mortalities, but 70% patients experienced perioperative complications, of which 25% were severe. Interestingly, the median OS from the diagnosis of metastatic disease in this study was 24.2 months, lower than the 30.2 months reported in the first study. Furthermore, the median OS from CRS/HIPEC was 16.1 months, while the median OS in the first laparoscopic HIPEC study for the patients who underwent gastrectomy after complete resolution of peritoneal disease was 29 months. The key difference in the 2017 study was that four out of the five patients who underwent gastrectomy had only cyt + disease, indicating that this group is likely to have better outcomes with regional therapy and gastrectomy compared to patients who have gross disease.

Collectively, these two trials have established the safety and feasibility of laparoscopic HIPEC and provide evidence about the importance of peritoneal disease control prior to gastrectomy/CRS in patient with limited PC. However, assessment of quality-of-life (QoL) outcomes is necessary to compare this intervention with other IP approaches, particularly outpatient NIPEC.

### Selected Retrospective CRS/HIPEC Studies

There have been several HIPEC retrospective studies reported in the literature, and hence, the studies described in this section is not an exhaustive list, but a selection of high-impact studies with a minimum of 100 patients.

The CYTO-CHIP study was a French multicenter retrospective propensity-matched cohort study that studied the survival outcomes after CRS/HIPEC (CRS-H) versus CRS alone (CRS-A) in patients with GC with either cyt + disease or limited PC [26]. Over the 25-year study period (1989–2014), 180 patients underwent CRS-H, and 97 patients underwent CRS-A. HIPEC regimens were heterogenous across study sites. Notably, only 35% of CRS-A patients received neoadjuvant systemic therapy compared to 62.8% of CRS-H patients (*p* < 0.001). Median PCI was 6 (range 0–25) for CRS-H and 2 (0–13) for CRS-A. Median OS and recurrence-free survival (RFS) with CRS-H were 18.6 and 11.6 months, compared to 11.4 and 7.6 months for CRS-A (*p* = 0.002 and 0.001). After propensity matching, both OS and RFS remained higher for CRS-H vs. CRS-A (18.8 vs. 12.1 months and 13.6 vs. 7.8 months, respectively). On multivariable analysis, CRS/HIPEC was associated with better OS in patients with PCI < 7. There are serious limitations to this study due to its retrospective nature and the prolonged study period during which the treatment paradigm of gastric cancer has changed significantly. Despite these limitations, the 20% five-year survival rate in patients who underwent CRS/HIPEC is a strong signal favoring CRS/HIPEC in selected patients with limited PC.

DGAV-HIPEC is a multicenter retrospective study in Germany that included 235 patients with GCPC and underwent CRS/HIPEC across 16 centers between 2011 and 2016 [27]. HIPEC regimens varied depending on medical center preference. Most patients received preoperative chemotherapy (*n* = 174, 74.0%). The cohort’s median PCI score was 8 (range 1–30), and almost half of the patients had a PCI ≤ 7 (*n* = 78, 46.4%). About 66% of patients achieved CC0-1 resection, but 66 patients (28.1%) of patients had no reported CC score. Median OS was 13 months, and 5-year OS was 6%. Similar to previous studies, lower PCI scores correlated with improved survival. The median OS for PCI 0–6, PCI 7–15, and PCI > 15 were 18, 12, and 5 months, respectively (*p* = 0.002). The authors concluded that only patients with low PCI followed by complete cytoreduction can achieve improved long-term survival.

### HIPEC — Ongoing Clinical Trials

While there are important findings in these key retrospective studies that support CRS/HIPEC, clinical trials with carefully planned study designs and deliberate patient selection are needed to better quantify the survival benefit of HIPEC and its role for GCPC. These are the current ongoing trials that aim to answer these questions (Table 4).

**Table 4:** Completed and ongoing randomized and non-randomized clinical trials of pressurized intraperitoneal aerosolized chemotherapy (PIPAC) in gastric cancer peritoneal carcinomatosis

Clinical Trial	Country	Study Design (size)	Study Treatment	Outcomes
Completed Nonrandomized Clinical Trials for PIPAC				
PIPAC-GA2 [62] Khomyakov et al. (2018)	Russia, single center	Phase II (n = 31)	SC (CAPOX) + PIPAC (IP cisplatin/doxorubicin) every six weeks	Median OS: 13 months Disease progression: 25.8% Major histologic response: 26.7% Complete histologic response: 60%
PIPAC C/D [63] Struller et al. (2018)	Germany, single center	Phase II (n = 25)	PIPAC (IP cisplatin/doxorubicin) every six weeks × 3 cycles	Median OS: 6.7 months Clinical benefit: 40% (complete, partial or stable) radiographic response; 26%
Ongoing Clinical Trials for PIPAC				
PIPAC VerONE [66, 67] Casella et al. NCT05303714	Italy, multicenter (2022–2028)	Phase III 1:1 randomization (n = 98)	Control: SC (FOLFOX) Study: SC (FOLFOX) + PIPAC (IP cisplatin/doxorubicin) every 2 cycles At restaging, if stable disease or response, then proceed with CRS/HIPEC	Primary: Secondary resectability rate Secondary: 3.5-yr OS, 3.5-yr PFS, 3.5-yr disease-related survival, histologic response, QoL, AE, cost effectiveness
Luksta et al. [68, 69] NCT05644249	Lithuania, multicenter (2022–2027)	Phase II, nonrandomized (n = 37)	Single arm: SC (FOLFOX) + PIPAC (IP cisplatin/doxorubicin) every 2 chemo cycles	Primary: Objective response rate (complete and partial response) Secondary: Median number of PIPACs, PIPAC characteristics, treatment morbidity, interval PCI, biochemical tumor response, QoL, PFS
SPECTRA [70] Hanna et al. NCT05318794	United Kingdom, single center (2023–2030)	Phase II, nonrandomized (n = 20)	Single arm: SC + PIPAC (IP cisplatin/doxorubicin)	Primary: feasibility and safety Secondary: tumor regression, treatment morbidity, QoL, 5-yr recurrence, 5-yr OS
PIPAC EstoK 01 [60, 61] Eveno et al. NCT04065139	France, multicenter (2020–2024) Discontinued due to high number of deaths in study arm; follow-up through 2024 with results to follow	Phase II 1:1 randomization (n = 66)	Control: SC alone Study: SC + PIPAC (IP cisplatin/doxorubicin) every 2 chemo cycles	Primary: 2-yr PFS Secondary: 2-yr OS, safety and tolerability, QoL, resectability rate

*PIPAC* pressurized intraperitoneal aerosolized chemotherapy, *SC* systemic chemotherapy, *CAPOX* capecitabine/oxaliplatin, *IP* intraperitoneal, *OS* overall survival, *FOLFOX* leucovorin/5-fluorouracil/oxaliplatin, *CRS* cytoreductive surgery, *HIPEC* heated intraperitoneal chemotherapy, *yr* year, *PFS* progression-free survival, *QoL* quality-of-life, *AE* adverse events, *PCI* peritoneal cancer index

The GASTRICHIP study (NCT01882933) is a French, prospective, multicenter, randomized phase III trial designed to test the benefits of adding HIPEC to curative surgery (gastrectomy with D1–D2 lymphadenectomy) and perioperative systemic therapy in patients with advanced GC (T3-T4) with positive lymph nodes and/or cyt+ [28, 29]. Patients will be randomized in 1:1 fashion intraoperatively after curative resection to either HIPEC with oxaliplatin (250 mg/m^2^) for 30 min vs. surgery alone. This study is limited to locally advanced GC with cyt+, without evidence of gross or macroscopic peritoneal metastases. The primary endpoint is OS from the day of surgery and secondary endpoints are RFS at 3 and 5 years after surgery, site of recurrence, morbidity, and quality of life (QoL). The overall accrual goal is 322 patients based on the estimated 5-year survival of 30% for the control arm and 45% for the experimental arm (HR 0.67) with a two-sided alpha of 5%.

One of the critically important, yet unanswered question in the management of GCPC is about the added survival benefit of CRS/HIPEC to standard of care systemic chemotherapy (SC). Although the GYMSSA trial, by Rudloff et al. in 2014, attempted to answer this question, the study closed prematurely due to poor accrual after enrolling only 17 of the 168 required patients [30]. This question will likely be answered by the PERISCOPE II study (NCT03348150), a phase III trial in which patients with T3–T4 GC with limited peritoneal dissemination (PCI ≤ 7) will be randomized to either standard of care SC alone vs. SC + CRS/HIPEC and NIPEC [23, 31]. Based on anticipated improvement in survival from 3 to 12 months in the experimental arm and 90% power, the authors intend to enroll 106 patients, with 53 in each arm. These study results will determine whether there is a survival benefit and cost effectiveness with CRS/HIPEC (IP oxaliplatin)/NIPEC (IP docetaxel) for patients with limited PC as compared SC alone.

Meanwhile, the National Cancer Institute (NCI) in the USA has an active single center, phase II study evaluating CRS/HIPEC (cisplatin 90 mg/m^2^ and MMC 10 mg/m^2^) for GCPC patients with limited peritoneal dissemination (PCI < 10) (NCT03092518) [32, 33]. This trial has completed patient accrual, but remains active for primary data collection, with study completion anticipated in 2027. Primary endpoint is OS, and secondary outcomes are RFS (intraperitoneal and extraperitoneal) and treatment-related morbidity. Initial preliminary results were recently reported combined with the laparoscopic HIPEC study from MD Anderson Cancer Center, which showed median OS of 14.4 months and RFS of 7.4 months after CRS/HIPEC [25, 34]. Despite the low PCI, the low RFS calls for further refinement of patient selection and consideration of other IP strategies including novel IP treatment regimens.

## Normothermic Intraperitoneal Chemotherapy (NIPEC)

Normothermic intraperitoneal chemotherapy (NIPEC) is administered through a subcutaneous peritoneal port in outpatient settings. It is often combined with systemic therapy and repeated multiple times typically in 3-week cycles based on patient response. The most commonly tested NIPEC agent in GCPC is paclitaxel (PTX). Due to its large molecular weight (853.9 g/mol) and lipophilic nature, PTX is largely retained in the peritoneal cavity, achieving high IP drug levels with extremely low systemic absorption [35–37].

### NIPEC Randomized Clinical Trials

Several investigators in Japan and China have studied the utility of normothermic intraperitoneal chemotherapy (IP) combined with systemic chemotherapy to treat GCPC (Table 1) [38–42]. The largest RCT of NIPEC to date is the PHOENIX-GC trial from Japan, in which patients with GCPC were randomized in a 2:1 ratio to either the experimental regimen (IP and IV PTX plus S-1 (IP; IP PTX 20 mg/m^2^ and IV PTX 50 mg/m^2^ on days 1 and 8 plus S-1 80 mg/m^2^ per day on days 1 to 14 for a 3-week cycle) or S-1 plus IV cisplatin (SP; S-1 80 mg/m^2^ per day on days 1 to 21 plus cisplatin 60 mg/m^2^ on day 8 for a 5-week cycle) [43].

Although the PHOENIX-GC study failed to show a statistically significant improvement in survival with IP PTX plus systemic chemotherapy, due to crossover to IP arm and imbalance between the groups with more patients with moderate to severe ascites randomized to IP arm, the exploratory analyses suggested possible clinical benefits of IP PTX. The 3-year survival rate was significantly better in IP/IV PTX + S1 arm compared to SP (21.9% vs 6%) [43]. There were also concerns about the statistical power to detect a benefit in the IP arm in this trial. The trial enrolled 183 patients, which was the planned sample size calculated according to a median survival time (MST) of 22 and 11 months for the IP and SP arms, respectively. The value of 22 months came from three previous studies that reported MSTs of 17.6, 20.6, and 22.5 months for the IP group [44–46]. However, the latter two studies enrolled a proportion of patients with positive peritoneal cytology without macroscopic PC, whereas the PHOENIX-GC trial only included patients with macroscopic PC. Because patients with macroscopic PC generally have a worse prognosis than those with cyt + only, the length of survival for the IP arm might have been overestimated, which resulted in the study being underpowered. Moreover, in the IP group of the PHOENIX-GC trial, only one of 122 patients had surgery. However, the surgical rate was 64% (*n* = 64/100) in the trial by Ishigami et al. that was used for calculation of sample size. Ishigami et al. also found that the MST was 30.5 months (95% CI, 23.6–37.7 months) and 14.3 months (95% CI, 10.0–17.8 months) in patients who underwent surgery and those who did not, respectively. This suggests that surgery after response to intraperitoneal and systemic chemotherapy may improve the overall survival of patients with gastric cancer with limited peritoneal metastasis.

There has been an additional phase II randomized trial with varying results. The INPACT study was a multicenter phase II trial in Japan which tested the survival benefit of immediate postoperative administration of IP vs. IV paclitaxel in patients who underwent surgical resection of advanced GC and had either cyt + or limited PC [47]. Patients in the IP arm received 40 mg/m^2^ of PTX and IV arm received 80 mg/m^2^ of PTX on postoperative days 0, 14, 21, 28, 42, 49, and 56. Systemic chemotherapy with S-1 alone or S-1 + cisplatin was started after completion of the PTX study treatment. There was no difference in OS or PFS between both groups. Needless to say, the approach used in INPACT study is widely deviant from the standard of care in the Western countries as upfront surgical therapy without neoadjuvant systemic treatment would be considered unacceptable in patients with stage IV GC. Furthermore, withholding systemic therapy for 2 months postoperatively in patients with untreated stage IV GC would be deemed unethical. It is important to recognize that bidirectional approaches with optimal preoperative systemic control are necessary to treat GCPC and IP therapy alone may not lead improved survival.

With the varying study designs and results from these phase II and phase III randomized trials, additional studies and trials are needed to better define the role and clinic impact of NIPEC for GCPC.

### NIPEC — Selected Non-randomized Clinical Trials

There have been multiple phase II nonrandomized trials (Table 3), investigating additional endpoints such as histologic response and IP conversion to achieving surgical resection were explored.

In Singapore, Chia et al. performed a phase II nonrandomized single arm study evaluating IP PTX (40 mg/m^2^ on days 1 and 8) with systemic chemotherapy (SC; XELOX) for a median of eight cycles for 44 enrolled patients, then performed outcomes analysis with a matched retrospective cohort that received SC alone [48]. The median OS was improved for patients who received IP PTX with SC to 14.6 months, as opposed to 10.6 months in the matched cohort (*p* = 0 0.002). PFS was improved as well to 9.5 months for IP PTX patients, compared to 4.4 months for SC alone (*p* < 0.001). Furthermore, the addition of IP PTX resulted in response allowing 13 patients (36.1%) to undergo CRS, improving survival even further to 24.2 months.

Kobayashi et al. tested the addition of IP PTX 20 mg/m^2^ administered on days 1, 8, and 22 combined with S-1 and cisplatin in 5-week cycles (*n* = 53). The median OS was 19.4 months (6.1–24.6 months), and PFS was 11.1 months (8.4–15.9 months) [49]. Notably, in both studies by Chia et al. and Kobayashi et al., surgical resection in a highly selected subset of patients (30–36%) who responded to IP paclitaxel as confirmed by negative peritoneal cytology and/or histologic response of the peritoneal nodules was associated with even better survival outcomes. Kobayashi et al. demonstrated OS of 42.1 months (34.9–43.5 months) and PFS of 18.1 months (14.8–29.3 months) in the sixteen patients (30%) that underwent surgical resection.

Similarly, in Japan, Shinkai et al. performed a single center phase II study that evaluated IP PTX with systemic chemotherapy (SC; S-1 with IV cisplatin/PTX) [50]. Seventeen patients were enrolled and underwent a median of 7 courses of SC with NIPEC PTX. Of these patients, 64.7% patients (*n* = 11/17) converted to negative cytology and underwent surgery. There were six remaining patients with persistent PC disease. Median OS was 32.8 months for the patients who underwent surgery after complete resolution of peritoneal disease, as compared to 12.9 months for the patients with persistent PC (*p* < 0.05). One- and 5-year survival were 82.4% and 23.5% for the entire cohort.

Though nonrandomized in nature, these phase II trials show promise for NIPEC as regional therapy in conjunction with SC to help improve survival as compared to SC alone. In addition, NIPEC has an emerging role as bridge to control and improve PC burden, allowing for the possibility of definitive surgery for select patients with GCPC.

### NIPEC — Selected Ongoing Clinical Trials

There are several ongoing single arm NIPEC phase II trials. We review a selection of ongoing trials in the USA, Brazil, and South Korea (Table 4).

The STOPGAP I is a single-center, single arm, phase II trial that is evaluating the benefit of iterative NIPEC PTX combined with systemic therapy in patients with GC with either cyt + or PC (NCT04762953) [51, 52]. Adult patients (18–75 years old) with gastric and GEJ adenocarcinoma with cyt + or PC detected by laparoscopy, laparotomy, or imaging and without evidence of other distant organ metastasis after the induction period of first-line systemic therapy are eligible for this study. Systemic therapy prior to enrollment is at the discretion of the treating physician and is chosen based on the biomarker profile of the tumor to maximize the tumor response and reduction in disease burden prior to enrollment on the protocol. The IP regimen consists of IP PTX 40 mg/m^2^ administered on days 1 and 8 combined with systemic 5-FU and leucovorin and IV PTX in three-week cycles for four cycles. Reevaluation with imaging and diagnostic laparoscopy is performed after the completion of four cycles. In patients with stable disease or response, IP treatment is continued beyond four cycles. In the subset of patients with PCI $\le$ 10 where complete cytoreduction is feasible, surgical therapy is offered. The primary endpoint is 1-year PFS, and the secondary endpoints are OS and patient-reported QoL measured by EuroQol-5D-5L.

Similar to the STOPGAP I study, Dias et al. have recently started a single center, single-arm phase II trial in Brazil evaluating the safety, tolerability, and peritoneal response rate of systemic chemotherapy with the addition of IP PTX (40 mg/m^2^) on days 1 and 8 (NCT05541146) [53, 54]. This study aims to enroll 30 GC patients with cyt + or PC (PCI ≤ 12). Systemic chemotherapy will be determined by the treating physician. After four cycles, patients with clinical and radiographic peritoneal response will undergo restaging laparoscopy. Patients with complete peritoneal response (negative biopsies and negative cytology) will be eligible for gastrectomy (conversion surgery).

The IPLUS study from South Korea is a phase I-II single center trial assessing IP paclitaxel in combination with systemic mFOLFOX for GCPC (NCT03618758) [55]. The phase I dose-escalation study that included 13 patients established the fixed dosing of IP PTX (60 mg/m^2^ over one hour) for phase II [56]. The primary endpoint for IPLUS phase II is 1-year OS. Secondary endpoints are 1-year PFS, toxicity, tumor response, and conversion to surgery.

## Pressurized Intraperitoneal Aerosolized Chemotherapy (PIPAC)

PIPAC was first introduced in Germany in 2014 as a novel alternative technique to deliver IP chemotherapy [57], particularly for patients who are not candidates for CRS/HIPEC. By nature, most of these patients have relatively higher PCI. The pressurized and aerosolized administration of chemotherapy promotes improved homogenous distribution and uptake throughout the peritoneum [57–59]. There are specific equipment (nebulizer) and training required for PIPAC. Furthermore, due to the aerosolization of chemotherapy, the OR staff must exit the operating room during the treatment, leading to some logistical considerations with the establishment of PIPAC programs.

### PIPAC Randomized Clinical Trials

The PIPAC EstoK 01 trial (NCT04065139) in France is a phase II randomized trial that sought to investigate the addition of PIPAC to systemic therapy for GCPC [60, 61]. The study arms are SC alone vs. SC with PIPAC (doxorubicin 2.1 mg/m^2^ and cisplatin 10.5 mg/m^2^ with flow rate 0.7 mL/s) and included GCPC patients with PCI > 8. The primary endpoint is 2-year PFS, with secondary endpoints including 2-year OS, safety and tolerability, QoL, feasibility of repeated peritoneal access for iterative PIPAC, and resectability rate. Unfortunately, this trial was discontinued after accruing 66 patients due to a high number of deaths in the study arm. The study follow-up and completion are anticipated in 2024, with results to follow.

### PIPAC- Selected Non-randomized Clinical Trials

There have been several clinical phase II trials focused on PIPAC for GCPC (Table 3). In Russia, Khomyakov et al. conducted the phase II PIPAC-GA2 trial for GCPC patients who received bidirectional chemotherapy with systemic XELOX alternating with PIPAC every 6 weeks until disease progression or death [62]. The analysis included 31 patients who underwent 56 PIPAC procedures. The mean PCI was 16, and the burden of disease was classified as low PCI ≤ 9, moderate PCI 10–20, and high PCI > 20. These patients tolerated treatment without any major complications; however, disease progression was noted in 8 (25.8%) patients. Of 15 patients with at least two PIPAC cycles, four patients demonstrated complete pathologic response, while five patients demonstrated major pathologic response. Median survival for this cohort was 13 months.

Meanwhile, in Germany, Struller et al. reported results of their phase II PIPAC C/D trial for 25 patients with recurrent GCPC treated with three courses of PIPAC with cisplatin and doxorubicin cycle every 6 weeks [63]. Mean PCI was 15.3 at presentation and dropped to 13.3 after PIPAC cycle 3. The primary endpoint was clinical benefit from PIPAC, which was seen in 10 patients (40%) based on radiographic response. There was complete radiographic response in one patient, while partial radiographic response was seen in two patients and seven patients had stable disease. When assessing histologic response, 9/25 patients (36%) had complete or partial histologic regression. There were no major adverse events and median OS was 6.7 months.

The results of these two clinical trials have provided preliminary data and positive signal for the use of PIPAC in the advanced GCPC. However, the closure of EstoK 01 phase II study has raised some concerns about the safety of PIPAC in advanced GCPC and results of that study will shed light about the subgroup pf patients in whom PIPAC may still be safe and feasible.

### PIPAC — Selected Retrospective studies

Retrospective studies from Germany [58] and France [64] discussed the use of PIPAC with low-dose cisplatin and doxorubicin at 6-week intervals, alternating with systemic chemotherapy. Alyami et al.’s study included 164 patients with unresectable PC from other origins (ovarian, colorectal, primary peritoneal) in addition to GCPC who underwent 164 PIPAC procedures. The authors found that PIPAC was safe and tolerable, often leading to improvement and resolution of PC symptoms [64]. The median PCI at presentation was 19, improving to 15 after three consecutive PIPAC treatments. Six patients were deemed resectable and underwent CRS/HIPEC. In their updated analysis, Alyami et al. analyzed the 42 patients with GCPC who underwent 163 PIPAC procedures [65]. Median PCI at presentation was 17, and median number of PIPAC treatment was three. In this cohort, six (14.3%) patients demonstrated response to PIPAC, and subsequently underwent CRS/HIPEC. OS was 19.1 months. Nadiradze et al. s retrospective study cohort consisted of 24 GCPC patients who underwent 60 PIPAC procedures [58]. Mean PCI at presentation was 16 ± 10. Notably, 50% of patients demonstrated histologic response to PIPAC therapy, with 25% of patients having complete histologic regression. Median OS was 15.4 months. The results of these retrospective studies are promising for these advanced GCPC patients.

### PIPAC Ongoing Clinical Trials

There are also several clinical trials ongoing for PIPAC for GCPC (Table 4). PIPAC VerONE (NCT05303714) is a multicenter, randomized phase III trial that aims to evaluate the effectiveness of bidirectional PIPAC with systemic chemotherapy for patients with limited peritoneal disease [66, 67]. Enrollment is limited to patients with limited peritoneal disease (cyt + and/or PCI ≤ 6). Patients will be randomized to systemic therapy with PIPAC (FOLFOX × 6 cycles + PIPAC (cisplatin and doxorubicin) every two cycles of FOLFOX) or systemic therapy (six cycles of FOLFOX) alone. Patients with disease progression will proceed to second-line therapy, while patients with stable disease or treatment response, and a surgical candidate will proceed to CRS. Those with R0 resection will also receive HIPEC. Based on anticipated 30% improvement in achieving resectability with the treatment arm and 80% power, the authors are targeting 98 patients with 49 patients in each arm. The results from this trial will provide the treatment benefits in achieving resectability, survival, recurrence, and QoL for patients with limited PC.

Meanwhile, in Lithuania, Luksta et al. recently introduced a similar phase II clinical trial assessing the efficacy and treatment response of systemic FOLFOX alternating with PIPAC (cisplatin and doxorubicin) for patients with GCPC [68, 69]. Unlike PIPAC VerONE, there is no PCI cutoff for this trial and GCPC patients are eligible irrespective of PCI. Enrollment began in 2022, with study completion anticipated in 2027 and planned enrollment of 37 patients. This trial in particular aims to assess objective response using RECIST criteria as well as other bioclinical criteria.

The new SPECTRA phase II PIPAC clinical trial at the Imperial College London has started enrollment in November 2023 and is estimated to be completed in 2030 [70]. This safety and feasibility trial is limited to GCPC patients with minimal peritoneal disease (cyt + or PCI ≤ 3). Three cycles of PIPAC (cisplatin and doxorubicin) will be interposed with systemic chemotherapy (determined by the treating oncologist). At restaging laparoscopic assessment, patients with negative peritoneal cytology and PCI of 0 will be candidates for gastrectomy with D2 lymphadenectomy. This trial will evaluate the efficacy of PIPAC and systemic therapy to achieve resectability as well as tumor regression and recurrence in patients with limited peritoneal disease.

## Conclusions

Regional therapies have become a critical part of the multimodal treatment of GCPC. Particularly, the recent advances in systemic therapy offer an exciting opportunity to improve peritoneal disease control using bidirectional approach and in select patients, perform CRS. Each regional therapy modality—HIPEC, NIPEC, and PIPAC—has shown varying degrees of promising results, with improvements in survival rates and peritoneal disease control. However, the importance of patient selection cannot be overemphasized, particularly when it comes to CRS. While older studies were less restrictive in offering CRS to patients with a wide range of peritoneal disease burden, it has irrefutably been shown that patients with greater PCI may not have a significant (disease-free) survival benefit. As such, current ongoing and new upcoming trials are more selective in the PCI inclusion criteria, including optimization of upfront systemic regimens to further reduce the PCI. These exciting trials will help guide and shape the treatment paradigm and improve outcomes for gastric cancer patients with limited peritoneal disease.

## Data Availability

No datasets were generated during and/or analyzed for this current review.
